# Biosynthesis and Function of Extracellular Glycans in Cyanobacteria

**DOI:** 10.3390/life5010164

**Published:** 2015-01-12

**Authors:** Jan-Christoph Kehr, Elke Dittmann

**Affiliations:** University of Potsdam, Institute for Biochemistry and Biology, Department of Microbiology, Karl-Liebknecht-Str. 24/25, 14476 Potsdam-Golm, Germany; E-Mail: editt@uni-potsdam.de

**Keywords:** cyanobacteria, exopolysaccharides, lipopolysaccharides, colony formation

## Abstract

The cell surface of cyanobacteria is covered with glycans that confer versatility and adaptability to a multitude of environmental factors. The complex carbohydrates act as barriers against different types of stress and play a role in intra- as well as inter-species interactions. In this review, we summarize the current knowledge of the chemical composition, biosynthesis and biological function of exo- and lipo-polysaccharides from cyanobacteria and give an overview of sugar-binding lectins characterized from cyanobacteria. We discuss similarities with well-studied enterobacterial systems and highlight the unique features of cyanobacteria. We pay special attention to colony formation and EPS biosynthesis in the bloom-forming cyanobacterium, *Microcystis aeruginosa*.

## 1. Introduction

**Figure 1:**
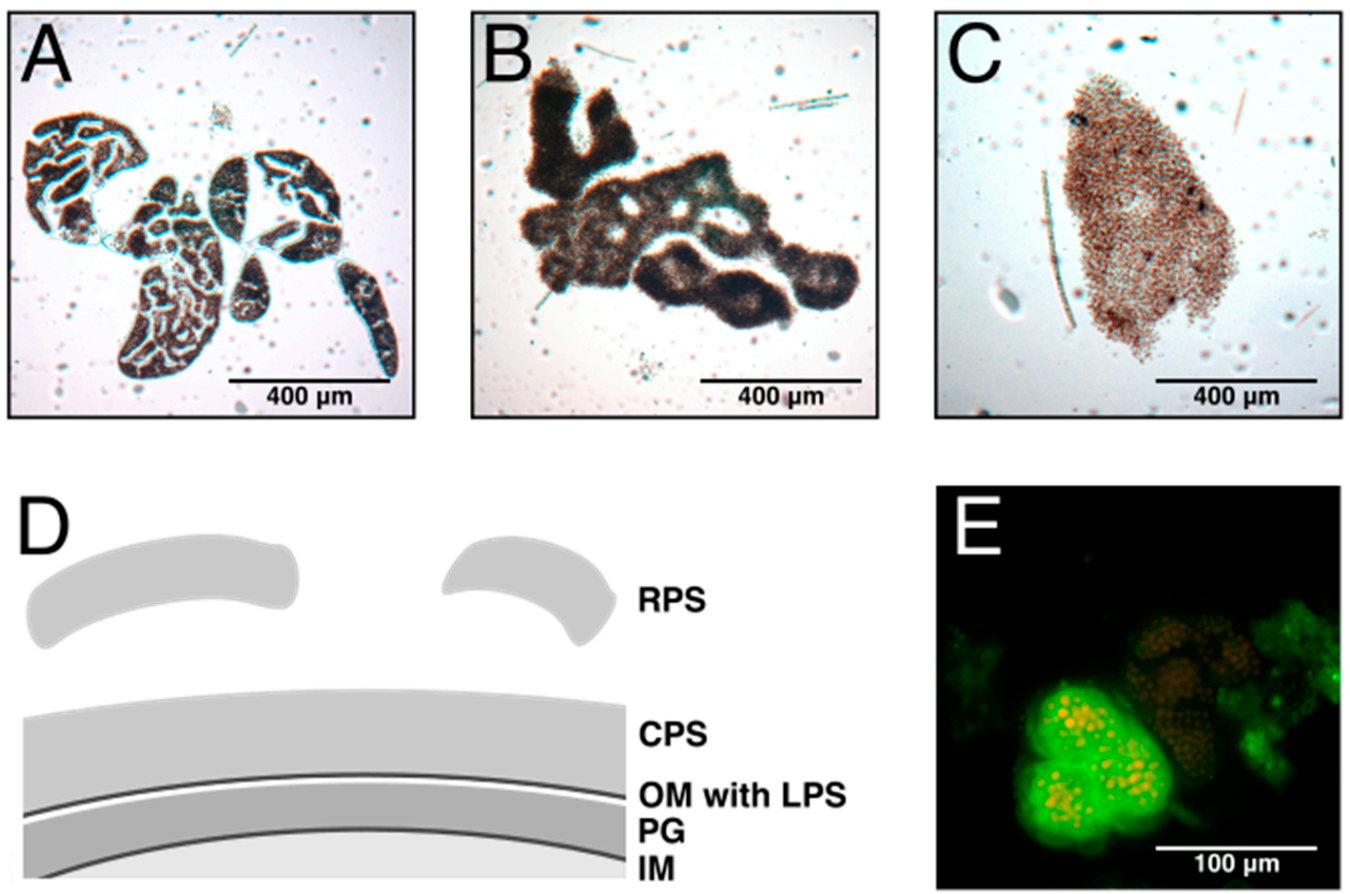
EPS in *Microcystis*. Light micrographs of characteristic morphotypes of (**A**) *M. wesenbergii*. (**B**) *M. aeruginosa* and (**C**) *Microcystis* sp. The colony morphology is determined by the mucilage embedding the cells. (**D**) The exopolysaccharides (EPS) are further classified as O-antigens of lipopolysaccharides (LPS) anchored in the outer membrane (OM), capsular polysaccharides (CPS), which are associated with the cell surface, and released polysaccharides (RPS), which are secreted to the culture medium without attachment to the producing cells. PG, peptidoglycan; IM, inner membrane. (**E**) The fluorescein isothiocyanate-labelled lectin, microvirin, bound to a *Microcystis* colony. Selective binding of MVN shows different exopolysaccharide composition in identical *Microcystis* morphotypes.

Cyanobacteria are a diverse group of photosynthetic bacteria that exhibit a wide range of morphological shapes. The phylum comprises species that are unicellular, grow in colonies or form true multicellular filaments. Many species produce high amounts of mucilage that is often used as criterion for species determination. Cyanobacteria of the genus *Microcystis*, as an example, occur in distinct colony morphotypes, which are differently shaped by the mucilage that embeds the cells (Figure 1A–C). This ubiquitous cyanobacterium frequently forms dense blooms (containing a mixture of morphotypes) in eutrophic freshwater lakes and represents a serious health threat due to the toxins produced by many strains [1]. Cyanobacteria may also change their extracellular glycan composition dynamically. A varying surface sugar composition was, for instance, shown for distinct differentiation steps of the symbiotic cyanobacterium, *N. punctiforme* [2]. Whereas these examples emphasize the importance of glycan structures in an environmental and physiological context, a systematic analysis is currently missing.

Most cyanobacteria are surrounded by a matrix of polymeric substance, which forms a protective boundary between the bacterial cell and the immediate environment [3]. The secreted material is referred to as extracellular polymeric substances and is mainly composed of complex heteropolysaccharides. Extracellular polymeric substances, as well as exopolysaccharides are both commonly abbreviated as EPS, which might cause some confusion. In this article, the term EPS will be exclusively used to refer to exopolysaccharides. EPS are attached to the cell surface as capsular polysaccharides (CPS) or delivered to the culture medium as released polysaccharides (RPS) (Figure 1D). The CPS can appear as a sheath, usually a thin, defined layer loosely covering cells or assemblies of cells, a capsule, a thick layer tightly associated with a single cell, or slime, which surrounds the cells, but does not form a distinct shape [3]. In addition, the cells are covered by lipopolysaccharides (LPS) anchored in the outer membrane. Although many studies addressed the questions of the composition and function of cyanobacterial extracellular glycans [4,5,6,7,8,9], knowledge of them is still limited compared to other bacteria. In fact, there is intensive research on the biotechnological exploitation of cyanobacterial EPS, which led to the elucidation of the monosaccharide composition and the physico-chemical properties of EPS from many strains [10]. Nevertheless, the discovered complexity of EPS makes complete structure elucidation difficult. Therefore, it is not a surprise that cellulose, which is a component of the extracellular matrix of several cyanobacteria of Sections I, III and IV and consists only of glucose, is among the best-characterized polysaccharides in cyanobacteria [11]. The high diversity of monosaccharide building blocks defines the unique properties of cyanobacterial EPS and clearly sets them apart from other bacteria [12].

Glycans are by far the most complex repeating biomacromolecules in biological systems, and their ability to encode information is tremendous. Other than linear oligonucleotides or peptides, glycans can form branched molecules, where branching can occur on several positions of a monosaccharide (typically three or four). Werz *et al.* [13] have calculated that a trimer allowing the incorporation of the 10 most frequently occurring mammalian monosaccharides can have 126,000 possible combinations, exceeding the possible diversity of a trinucleotide (64) or tripeptide (8000) by far. Additionally, modifications, like methylation, acetylation and the addition of sulfate or pyruvate groups, can enhance diversity further [6]. Considering the high structural diversity that can be achieved by even a small number of building blocks, it is difficult to infer the properties or functions from just the monosaccharide composition of a polysaccharide.

In this review, we would like to present an overview of the current state of glycan research in cyanobacteria covering the composition and physico-chemical properties, the biosynthesis, as well as the function of extracellular polysaccharides.

## 2. Composition and Structure of Cyanobacterial EPS

Cyanobacterial exopolysaccharides consist of repeating units built from monosaccharides that result in molecules several hundred kDa in size, with the largest molecules reaching a molecular weight of 2 MDa [14]. The repeating units are typically made from five to eight monosaccharides, but few cyanobacteria exhibit a much higher complexity with repeating units comprised of up to 15 monosaccharides (an extensive summary is given in [12]). This is a unique feature of cyanobacteria, since other microorganism usually possess carbohydrate polymers that contain up to four monosaccharide building blocks only. Exopolysaccharides of cyanobacteria contain various hexoses (fructose, galactose, glucose and mannose), pentoses (arabinose, ribose and xylose) and deoxyhexoses (fucose and rhamnose), as well as the acidic sugars, glucuronic and galacturonic acid. Further modifications include methylation, sulphatation, acetylation and the introduction of peptide moieties. The presence of acidic sugars is only rarely observed in the EPS of other Gram-negative bacteria. Together with commonly occurring sulfate groups, acidic sugars are responsible for the anionic nature of cyanobacterial EPS, which enables the ability for metal sequestration [10]. Due to their polyanionic nature, cyanobacterial exopolysaccharides form hydrated gels.

## 3. Unique Features of Cyanobacterial Lipopolysaccharides

Lipopolysaccharides provide a permeability barrier to large, negatively-charged and/or hydrophobic molecules and contribute to the structural properties of the cell envelope [15]. LPS is an important surface structural component of Gram-negative bacteria and covers ~75% of the surface area of the outer membrane. It is a tripartite molecule consisting of lipid A, which is embedded in the outer membrane, a conserved glycan core attached to the lipid and a variable O-antigen extending the glycan core [16]. While in most bacteria, the LPS core is conserved and contains 3-deoxy-d-*manno*-octulosonic acid (KDO) and heptoses, these sugars are absent from cyanobacteria [9,17]. Additionally, lipid A is devoid of phosphate groups. Instead, galacturonic acid is linked to lipid A, introducing a negative charge [18]. The O-antigen is composed of glycans that show a high variability within and between species. The structural heterogeneity of the O-antigen portion confers versatility and adaptability to bacteria that are exposed to variable environmental conditions. The presence or absence of O-antigen defines either a smooth or rough phenotype [19].

## 4. Biosynthesis of Extracellular Glycans

The biosynthesis of exopolysaccharides was shown to occur through very similar mechanisms throughout the bacterial kingdom. Three major biosynthetic routes are known, which share some similarities, but they are also characterized by fundamental differences. These pathways are distinguished by the enzymes responsible for the translocation of the polysaccharide or repeating units through the inner membrane. In a Wzx/Wzy-dependent system, repeating units are transferred to the periplasmic side of the inner membrane by a flippase (Wzx), where the final assembly of the nascent polysaccharide happens at the Wzy protein [20]. In an ABC transporter-dependent system (Wzm/Wzt-dependent), the polysaccharide is completely synthesized inside the cell before it is released through the ABC transporter, Wzm/Wzt [21]. In the third pathway (synthase-dependent), whose mechanistic details are not yet understood, a single enzyme that serves both as polymerase and an exporter facilitates the export [22].

**Figure 2:**
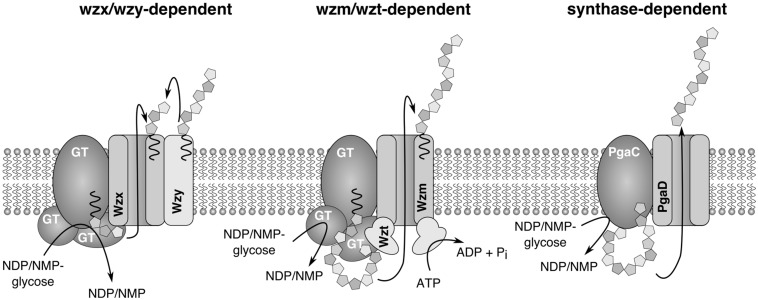
Models of three distinct EPS biosynthesis routes in *E. coli*. In the Wzx/Wzy-dependent system, repeat units are synthesized at the cytoplasmatic site of the inner membrane and translocated into the periplasm by the flippase, Wzx. Wzy assembles the repeat units to the final polysaccharide. In the Wzm/Wzt system, the complete polysaccharide is synthesized at the inner membrane and exported by the ABC transporter Wzm/Wzt. In the synthase-dependent system, chain elongation is directly linked to transport, but the details are unknown.

Several biosynthetic routes in Gram-negative bacteria were elucidated, and the corresponding genes were identified [20,21,23,24,25,26]. Commonly, the genes involved in exopolysaccharide biosynthesis are clustered, and the nomenclature is consistent among different species, while in most cyanobacteria, the genes are clustered in smaller units or even orphaned and dispersed over the whole chromosome. Additionally, automated genome annotation led to misannotations and an inconsistent naming. Therefore, the detailed description of glycan biosynthesis below will follow the general scheme for Gram-negative bacteria and highlight differences described in cyanobacteria. Since only a few cyanobacterial pathways have been analyzed in detail, it is not clear if the mechanisms apply to all cyanobacterial genera. Capsular polysaccharides in *E.coli* are classified into four groups, where Groups 1 and 4 and Groups 2 and 3 share similar biosynthesis routes [25]. The proteins that facilitate initiation and transport of the glycopolymer are conserved and present in all members of each group, while the presence of serotype-specific enzymes providing monosaccharide building blocks and glycosyltransferases linking these to the growing polysaccharide chain determines the specific composition of the glycan [25]. Group 1 and 4 polysaccharides are assembled by the Wzx/Wzy systems, and Group 2 and 3 glycans depend on the Wzt/Wzm (ABC transporter-dependent) system (Figure 2). In both cases, biosynthesis is initiated at the cytosolic face of the inner membrane at an integral membrane glycosyltransferase by the transfer of the first building block to a lipid carrier. The following steps differ significantly between both systems. In the Wzx/Wzy system, individual repeating units are assembled and transferred to the periplasmic side of the inner membrane by the flippase Wzx and are subsequently linked by the Wzy protein to the growing polysaccharide chain. This process further requires the integral membrane protein Wzc and the associated phosphatase Wzb (Figure 2). Finally, the nascent polymer is translocated through the outer membrane by the channel Wza. In contrast, in the ABC transporter-dependent Wzt/Wzm (*kpsT*/*kpsM*)-dependent system, polysaccharides are completely assembled at the inner cytoplasmic membrane and secreted by the ABC transporter, Wzt/Wzm. Later, a synthase-dependent pathway was discovered, in which the export is directly linked to polysaccharide synthesis [27]. This type of polysaccharide biosynthesis was shown to be involved in the synthesis of poly-β-1,6-*N*-acetyl-d-glucosamine encoded by the *pgaABCD* gene cluster [28] and cellulose encoded by the *bcsABZC* gene cluster [29] in *E. coli*.

To our knowledge, there are only a few studies that experimentally address exopolysaccharide biosynthesis and export in cyanobacteria [30,31,32]. Based on mutational and bioinformatic studies in *Synechococcus elongatus* PCC 7942, a model similar to the enterobacterial Wzm/Wzt system was proposed for the biosynthesis of the O-antigen part of lipopolysaccharides. It was further assumed that lipopolysaccharide biosynthesis occurs exclusively through this pathway, because orthologues of *wzx* and *wzy* were not found in *S. elongatus* [32]. In *Synechocystis* PCC 6803, ORFs encoding Wzm/Wzt-type ABC transporters *(slr0977*, *slr0982*, *slr0574* and *sll0575*) were shown to be involved in EPS synthesis. Mutants of these proteins showed an altered EPS pattern, but the compositions of O-antigen was unaffected, corroborating that other genes are responsible for the synthesis of the polysaccharide part of LPS [30]. Another study showed that ORFs *sll0923, sll1581* and *slr1875*, which encode proteins similar to components of the Wzx/Wzy pathway, are involved in CPS synthesis in *Synechocystis* PCC 6803 [31]. The biosynthesis of cellulose in *Thermosynechococcus vulcanus* occurs via a synthase-dependent pathway, which was confirmed by gene disruption of the putative cellulose synthase gene, *TvTll0007* [33].

**Figure 3:**
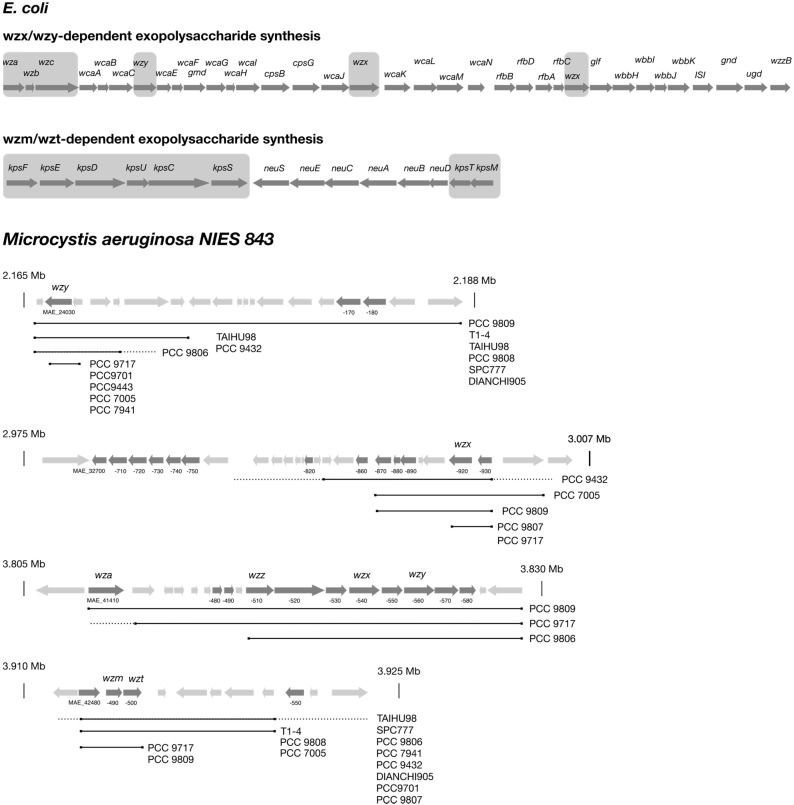
Genetic organization of EPS gene clusters in *E. coli*, as well as a representation of the loci harboring homologues of EPS core genes in the complete genome of *Microcystis aeruginosa* NIES 843 and draft genomes of other *Microcystis* strains. The typical organization of Group 1 and 4 and Group 2 and 3 capsule biosynthesis clusters is depicted for *E. coli* (**top**). Grey boxes highlight the conserved core genes. Genes encoding glycosyltransferases and precursor synthesis vary depending on the serotype. Wzx/Wzy and Wzm/Wzt gene homologues in *Microcystis* are shown in their genetic background (**bottom**). Additional genes putatively involved in EPS biosynthesis are shown in dark grey, and genes encoding unrelated functions are shown in light grey. The locus tags of relevant genes are depicted below. Solid black lines indicate similar sequences in *Microcystis* strains listed on the right end. Dotted lines represent upstream or downstream sequences, which do not show homology to corresponding flanking regions in NIES 843, and no line indicates missing sequence information due to unfinished genome status.

Nevertheless, many cyanobacterial genome sequences were added to the databases in recent years, which offers the opportunity to systematically screen for genes involved in glycan synthesis. We used query sequences for conserved genes, as well as serotype-specific glycosyltransferases from *E. coli* to identify putative EPS genes in *Microcystis aeruginosa* NIES 843 (Figure 3). Indeed, the presence of a Wzm/Wzt system could be confirmed with identities of 32% and 41% compared to the *E. coli* enzymes, although the genes are not embedded in a gene cluster harboring further glycan biosynthesis genes. Putative *wzx* and *wzy* genes could be identified, as well, but these showed only partial (~40%) coverage and low (~27%) identity to the *E. coli* query sequences. However, glycosyltransferases and sugar epimerases were identified in direct or close vicinity, implying that these genes might be involved in the synthesis of exopolysaccharides. In addition, methyltransferases and sulfotransferases were identified, which may facilitate further modifications of the glycans. Furthermore, several glycosyltransferases spread all over the chromosome were found, many of which could not be assigned to EPS synthesis based on their genomic context. Previous studies showed that the sequence identity of Wzx and Wzy orthologues in different microorganisms is very low, and thus, BLAST searches with query sequences from distant species might fail to identify Wzx and Wzy proteins if search parameters are not adjusted properly [34,35]. We also tried to estimate the level of conservation of the putative EPS synthesis loci in other *Microcystis* genomes [36,37,38,39] by looking for homologues of the conserved pathway enzymes (Figure 3). Apparently, at least one *wzm/wzt* and *wzx/wzy* system could be identified in each strain, with some strains possessing multiple *wzx/wzy* systems. Furthermore, some variations in the genetic neighborhood were observed, although upstream and downstream regions of *wzm/t/x/y* genes were frequently not assessable due to the unfinished status of most genomes. However, a mosaic-like distribution, rather than a clustered organization, of genes implicated in EPS biosynthesis over the whole chromosome might indicate frequent recombination, which contributes to strain-specific glycan structure diversification.

## 5. Overview of Lectins in Cyanobacteria

Lectins are carbohydrate-binding proteins that recognize and attach to complex glycans. They often contain two binding sites or form oligomers effectively multiplying the number of binding sites, which makes them key players in interaction and recognition processes [40]. Though they are usually not glycosylated, they are important factors in glycan-mediated processes and, thus, must be covered by this article. Lectins show high specificity and can even discriminate between oligosaccharides built from the same monosaccharides, but linked by different glycosidic bonds [41]. Few cyanobacterial lectins have been described so far (Table 1), and most of them exhibit specificity to mannose-rich glycans (mannan). Mannan is the major glycan on the envelope of HIV, and some other viruses and cyanobacteria have been intensively screened for HIV entry inhibitors during the last decade [42]. Thus, the bias towards mannan-binding lectins does not necessarily reflect an abundant occurrence of mannan oligosaccharides in cyanobacteria. The interest in mannan-binding lectins led to an extensive biochemical characterization providing specificity, affinity and structural data for most of the isolated proteins, but virtually no information on the biological role of these lectins is available. Furthermore, many lectins contain signal peptides implying an extracellular function and association with cell surface carbohydrates. The lectin, microvirin (Mvn), from *Microcystis aeruginosa* PCC 7806, was shown to bind to LPS of the producing cells and was proposed to be involved in colony formation [43]. The fluorescently-labelled lectin could differentiate distinct *Microcystis* morphotypes in lectin binding analyses, thereby demonstrating the diverging glycan composition (Figure 1E). A lectin from *M. viridis* was detected only in a stationary phase culture grown without aeration [44,45].

**Table 1:** Cyanobacterial lectins with specificity for high-mannose glycans.

Lectin	Organism	Reference
microvirin (MVN)	*Microcystis aeruginosa* PCC 7806	[44,46]
cyanovirin-N (CV-N)	*Nostoc ellipsosporum*	[47,48]
scytovirin (SVN)	*Scytonema varium*	[49]
*Oscillatoria agardhii* agglutinin (OAA)	*Oscillatoria agardhii*	[50,51]
*Microcystis viridis* lectin (MVL)	*Microcystis viridis*	[45]
MAL	*Microcystis aeruginosa* M228	[52]

Lectins also represent a useful tool for the analysis of glycans. *In situ* hybridization with labelled lectins of known specificity can be used to characterize biofilms and to determine the presence of certain polysaccharide types [53,54]. Data thus obtained can provide information of the composition and types of glycosidic linkages without a time-consuming and elaborate chemical analysis.

## 6. The Role of Exopolysaccharides in Cyanobacteria

Many functional assignments of cyanobacterial exopolysaccharides are related to their physico-chemical properties and describe their role in metal-binding, scaffolding in biofilms or as diffusion barriers [55], while the knowledge of glycans as specificity mediating agents in biological interactions is scarce compared to that of other bacteria.

### 6.1. Colony Formation

The involvement of EPS in colony formation is evident for the species, *Microcystis* (Figure 1A), and several aspects of colony formation have been addressed in numerous studies [56,57,58,59,60,61]. The ability to form colonies seems to be beneficial in the natural environment, but *Microcystis* isolates cultivated in the laboratory under axenic conditions lose the ability to form colonies after a short period of time. Interestingly, the amount of EPS produced by laboratory strains was strongly reduced compared to fresh colonial growing isolates, which contained up to 10-fold higher amounts of EPS [62]. Various biotic and abiotic factors that could trigger EPS production were identified. It was shown that inter-species interactions could promote EPS production. Grazing pressure through flagellates led to the increase of EPS secretion [59], and cocultivation of an axenic culture of *Microcystis* with heterotrophic bacteria isolated from *Microcystis* field sample colonies reconstituted colony formation and stimulated EPS production [58]. Some studies report a correlation between toxin (microcystin) production and colony size [63], and indeed, the addition of microcystin led to increased colony size and EPS production, as well as induction of polysaccharide synthesis genes [56]. The addition of high, but not growth-inhibiting, concentrations of Ca^2+^ ions had a similar effect, which was attributed to the matrix stabilizing properties of divalent cations or the interactions with lectins, which often depend on Ca^2+^ to bind glycans [62]. Furthermore, the production of exopolysaccharides is negatively correlated with the specific growth, which might explain why the ability to form colonies is lost during laboratory culturing, where environmental growth rates are exceeded [64].

### 6.2. Symbiosis

A role of specific extracellular glycans in the recognition process of symbiotic partners was demonstrated for a broad number of phyla [65,66,67]. A dialogue of the partners involving glycan structures on one side and lectins on the other side is commonly anticipated [68]. Nitrogen-fixing heterocystous cyanobacteria are associates in manifold symbioses with plants and fungi. The infection process is facilitated by the differentiation of infectious motile hormogonia [69]. Lectins were applied to characterize the changing sugar composition at transition states between distinct developmental stages of *Nostoc punctiforme*. Schüssler *et al.* [2] could provide evidence that the differentiation from hormogonia to non-motile primordia correlates with the disappearance of β-d-galactosyl and α-d-fucosyl sugars and the appearance of a large amount of α-d-mannosyl or α-d-glycosyl sugars in the extracellular slime. Specific lectins correlated with the *Anabaena azollae* symbiosis. Only symbiotic *Anabaena azollae* strains contained a hemagglutinating factor that was not present in free-living *Anabaena azollae* strains. Antigenic cross-reactivity studies suggested a possible significance of common antigens of *Azolla* and *Anabaena azollae* and the involvement of a lectin [70]. The cyanolichen, *Peltigera canina*, was demonstrated to secrete an arginase with a lectin activity specifically recognizing a sugar receptor of the pre-symbiotic *Nostoc* cell surface, apparently using a polygalactosylated urease as a cell wall ligand [71]. Lectin secretion by the *Nostoc* host, *Leptogium corniculatum*, was also demonstrated, and the lectin was assumed to be a factor in recognition of a compatible host [72]. Although the composition of involved glycan structures is not fully understood, there is compelling evidence that glycans and lectins mediate the interaction of cyanobacterial symbionts and their hosts and act as specific recognition factors.

### 6.3 LPS Are Receptors for Cyanophages

Little is known about the specific role of cyanobacterial LPS, while in other bacteria, several examples emphasize their importance in intercellular interactions. In Gram-negative bacteria, the O-antigen is an important virulence factor of pathogenic bacteria, enabling infection, and it is required to establish successful symbiosis during host-microbe interactions [67]. In addition, bacterial surface structures can serve as receptors for bacteriophages. Besides surface-exposed proteins, like transporters and flagella, LPS are common targets for phage adhesion in bacteria [73]. Although many marine and freshwater cyanophages were described in recent years [74,75,76], little is known about the route of infection, but it can be assumed that phage infections in cyanobacteria follow the mechanisms established in other bacteria. Evidence for the involvement of LPS in phage adsorption was first provided for the cyanophage AS-1 infecting *Anacystis nidulans* [77]. Later, LPS were identified as the target of the phages A-1 and A-4 in *Anabaena* PCC 7120, because mutants with defective O-antigen synthesis became resistant against these phages [78]. Interestingly, the same mutant showed an aberrant heterocyst development, suggesting a role of LPS in cell differentiation.

### 6.4 EPS and Environmental Stress

Exopolysaccharides are implicated in a variety of stress conditions imposed by hazardous stimuli in the environment, as they constitute a physical barrier enveloping the cell. EPS production was increased by salt stress in *Synechocystis* strains [79], and *Synechocystis* mutants defective in EPS production were less tolerant to elevated salt concentrations [31]. The reports on the metal binding ability of cyanobacterial EPS and their biotechnological potential are numerous (a comprehensive review is given in [10]), but little is known about the biological significance. Jittawuttipoka *et al.* [31] could show that EPS offers protection against heavy metal stress (Co^2+^ and Cd^2+^), but can also contribute to the response to iron starvation. Investigation of photosynthetic biofilms from mine tailings that are rich in toxic metals showed that EPS production occurs in response to metal exposure [80]. However, the limited data do not allow drawing a general conclusion about the role of EPS in metal homeostasis. EPS may also play a role in retaining trace metals under conditions where the availability of these is limited, like in marine environments.

Several studies have reported an implication of EPS in UV-light adaptation. In *Nostoc commune*, EPS production was induced upon UV irradiation [81], and UV-absorbing of d-galacturonic acid, a common component of cyanobacterial EPS, might be responsible for the protective effects of exopolysaccharides [82]. In addition, EPS provide a compartment for UV-light absorbing compounds, like mycosporine-like amino acids (MAAs) or scytonemin, which are released by some cyanobacteria and accumulate in the extracellular matrix [81,83,84]. Some of the MAAs are glycosylated, which may be important to be retained in the mucilage of the producer [83,85]. In *Microcoleus* species, a desert-crust inhabiting cyanobacterium, EPS were shown to be important to reduce UV-induced ROS generation; although a precise mechanism was not provided [86].

Apart from the regular exposition to high UV irradiances, terrestrial cyanobacteria, like *Nostoc* species, are frequently exposed to desiccation [87], and the extracellular matrix plays a crucial role in adapting to these conditions. EPS can stabilize cells during the air-dried state and prevent membrane fusions [88]. Tamaru *et al.* [89] could show that O_2_ evolution in *Nostoc commune* was impaired after the removal of EPS and desiccation treatment, while cells with intact EPS showed normal O_2_ evolution. Furthermore, it was shown that EPS conferred heat resistance.

In *Thermosynechococcus vulcanus*, cellulose production was induced in response to low temperature and light illumination. The release of cellulose triggered cell aggregation as a means of physiological acclimation to avoid light stress, which could be reversed by cellulase treatment [33].

Metabolic imbalances can also represent a challenge for cyanobacteria, and exopolysaccharides can serve as a carbon sink if the C/N balance is shifted towards excess carbon. Otero and Vincencini [90] could induce either nude or capsulated cells in diazotrophic grown *Nostoc sp.* PCC 7936, depending on the availability of carbon.

## 7. Conclusions

Cyanobacterial exopolysaccharides feature some unique properties, like the high content of acidic sugar moieties or the complexity of their repeating unit building blocks. Tremendous progress has been made in the characterization of EPS, but studies on the biological function of EPS are still limited in number. In particular, the role of glycans in recognition processes has been rarely addressed, although several reports suggest an important participation of EPS in intra- and inter-species glycan-mediated interactions. Especially, the spatial and temporal dynamics of EPS production in cyanobacterial interactions, like colony formation or the interplay between host and cyanobiont during symbiosis, are not fully resolved. The large number of cyanobacterial genomes sequenced in recent years offers the opportunity to assess the EPS diversity at the molecular level and provides a basis for future studies in order to elucidate the function of glycans in cyanobacteria.
